# *Coxiella burnetii* replicates in *Galleria mellonella* hemocytes and transcriptome mapping reveals *in vivo* regulated genes

**DOI:** 10.1080/21505594.2020.1819111

**Published:** 2020-09-24

**Authors:** Andrea Kovacs-Simon, Georgie Metters, Isobel Norville, Claudia Hemsley, Richard W. Titball

**Affiliations:** a College of Life and Environmental Sciences – Biosciences, University of Exeter, Exeter, UK; b CBR Division, Defence Science and Technology Laboratory, Porton Down,Salisbury, UK

**Keywords:** *Coxiella burnetii*, *Galleria mellonella*, T4SS, virulence, transcriptome profiling

## Abstract

Larvae of the greater wax moth (*Galleria mellonella)* are susceptible to infection with *C. burnetii*, an obligate intracellular bacterial pathogen. We show that bacteria are found in hemocytes after infection, and occupy vacuoles which are morphologically similar to *Coxiella*-containing vacuoles seen in infected mammalian phagocytes. We characterized the infection by transcriptome profiling of bacteria isolated from the hemocytes of infected larvae and identified 46 highly upregulated genes. The encoded proteins are predicted to be involved in translation, LPS biosynthesis, biotin synthesis, scavenging of reactive oxygen species, and included a T4SS effector and 30 hypothetical proteins. Some of these genes had previously been shown to be upregulated in buffalo green monkey (BGM) cells or in mice, whilst others appear to be regulated in a host-specific manner. Altogether, our results demonstrate the value of the *G. mellonella* model to study intracellular growth and identify potential virulence factors of *C. burnetii*.

## Introduction

*Coxiella burnetii* is the etiological agent of Q fever, a disease of humans which is globally distributed [1]. Q fever in humans can occur in several forms, including disease with a poor prognosis [2]. Q fever is a zoonotic infection and wild and domestic livestock such as cattle, sheep, and goats are common hosts [3,4].

*C. burnetii* can only replicate at low pH (~pH 4.5) and has a unique lifestyle including adaption to reside in the acidic phagolysosome-like compartment. Unlike many other pathogens, *C. burnetii* does not actively manipulate early trafficking events to avoid lysosomal fusion. Rather, the bacteria enter host cells by the endocytic pathway and then reside and replicate in *Coxiella*-containing vacuoles (CCVs) [5], after they fuse with lysosomes [6]. The CCV interacts with autophagosomes, and as the bacteria replicate, the CCV can enlarge to occupy much of the infected cell [5,7]. Little is known about the mechanisms of virulence of *C. burnetii* and an improved understanding of virulence mechanisms is essential for the development of diagnostics, vaccines, and therapeutics. There is evidence that lipopolysaccharide (LPS) [8,9] is a virulence factor. Also, type I, II, and IV secretion systems are present in *C. burnetii* [10] and there is evidence that the Dot/Icm type IV system plays a role in disease [11]. Endosome acidification activates the type IV system [12] and it is estimated that the genome may encode over 130 type IV effectors [7,13]. However, only some effectors have confirmed roles in virulence and their functions have been elucidated [6]. For example, Cig57 interacts with the clathrin accessory protein FCHO2, promoting formation and maturation of the CCV [6]. CirA can serve as a GTPase-activating protein for RhoA [14]. CvpB appears to play a role in homotypic fusion of CCVs [15]. Interestingly, comparative genome analysis has revealed variations in the repertoire of T4SS effectors in different strains [7,11,16–18], including plasmid-encoded effectors [19].

Until recently, research on *C. burnetii* was hampered by the inability to grow the bacterium axenically. Ground-breaking work in 2009 [20] resulted in the formulation of a complex synthetic medium (ACCM-2, pH 4.75) that supported growth of *C. burnetii* outside of host cells. There have also been advances by developing *Galleria mellonella* (wax moth) larvae as an infection model for Q-fever [21]. *G. mellonella* larvae are now widely used as infection models for a range of bacterial and fungal pathogens [22,23], and we have recently reviewed the advantages and disadvantages of this insect model for *Coxiella* research [24]. In brief, the larvae can be incubated at 37°C, allowing the expression of temperature-regulated virulence genes. A defined infection site and the ability to challenge larvae with precise doses allow the 50% lethal dose (LD_50_) to be calculated. This allows the virulence of mutants or the efficacy of antimicrobial compounds, to be compared. *G. mellonella* possess specialized phagocytic cells (hemocytes) which, like neutrophils of mammals, show lectin-mediated phagocytosis and kill microorganisms via a respiratory burst [25]. Hemocytes possess Toll-like receptors and receptor binding triggers signaling via an NFκB-like pathway [25]. Therefore, the complex interplay between host and pathogen can be captured in a way that is not possible in cell culture infection systems [21]. The *G. mellonella* infection model has been used to investigate the relative virulence of naturally occurring *C. burnetii* isolates [26], and T4SS mutants, which are attenuated in mammalian cell lines, are also attenuated in *G. mellonella* [21,27]. The model has been used to test antibiotic efficacy and Norville *et al*. showed that larvae dosed with doxycycline, the recommended treatment for Q fever, showed delayed mortality [21].

In this study, we set out to further characterize the *G. mellonella* infection model, and to map the transcriptome of *C. burnetii* during infection of *G. mellonella* larvae. This has allowed us to identify genes that may play roles in infection and which could now be investigated further as targets for diagnostics, prophylactics, or therapeutics.

## Materials and methods

### Bacterial strains and culture conditions

*C. burnetii* Nine Mile phase II clone 4 (NMII, RSA439) was used in this study and was cultured axenically in ACCM-2 liquid medium [28]. Cultures were grown in T25 or T75 tissue-culture flasks (Nunc EasyFlask, Thermo Scientific) containing 6 ml or 25 ml medium and incubated at 37°C in 5% CO_2_ and 2.5% O_2_ in a MG500 Anaerobic Workstation (Don Whitley Scientific Limited). Manipulations of *C. burnetii* were carried out in a class I microbiological safety cabinet in a Biological Safety Level 2 (BSL2) laboratory.

### *Enumeration of* C. burnetii

Genome equivalents (GE) of *C. burnetii* were determined during growth in ACCM-2 medium and in infected *G. mellonella* using real-time PCR targeting the *com1* gene, as described by Norville *et al*. [21]. Generation times were calculated during the exponential phases of growth in ACCM-2 (day 1–4) or in *G. mellonella* (day 1–2) using the formula; doubling time = duration * log (2)/log (final concentration) – log (initial concentration).

### *Infection of* G. mellonella

*G. mellonella* were purchased from Biosystems Technology Ltd. *C. burnetii* cultures were adjusted to OD_600_ of 0.2 in PBS (equal to approximately 1 × 10^8^ GE/ml) and quantified by qPCR. Groups of 10–30 larvae were infected with *C. burnetii* (10 μl in PBS) into the upper left proleg and incubated at 37°C. Control larvae were injected with 10 μl sterile PBS. Unless otherwise indicated, larvae were challenged with 1 × 10^6^ GE of *C. burnetii*.

### *Growth in* G. mellonella

qPCR was performed on isolated hemocytes or larvae homogenates. For hemocytes, hemolymph was extracted by aseptically removing the bottom 2 mm of larvae and draining into a microcentrifuge tube. The hemolymph was centrifuged at 500 x g 4°C for 5 minutes to separate hemocytes before re-suspension in sterile PBS. For whole larvae homogenates, larvae were homogenized with 1.4 mm ceramic beads in a Precellys 24 homogenizer (Bertin Instruments Ltd).

### Visualization of infected hemocytes

Hemocytes were visualized using a Jeol JEM-1400 transmission electron microscope (TEM). At 3 days postinfection, hemocoel was extracted as described above into a microcentrifuge tube containing a small amount of phenolphetourea (PTU) to prevent melanization. Hemocytes were fixed overnight with 2% glutaraldehyde, 2% paraformaldehyde, 0.1 M sodium cacodylate pH 7.2 before embedding in 2% (w/v) agarose. Duplicate 10 minute washes were performed with 0.1 M sodium cacodylate pH 7.2 before fixation with 1% (w/v) osmium tetroxide, reduced with 1.5% (w/v) potassium ferrocyanide in 0.1 M sodium cacodylate pH 7.2. Fixed samples were embedded in a durcupan resin prior to sectioning and visualization.

### *Infection and processing of* G. mellonella *for transcriptomics*

*C. burnetii* cells from 10 ml of the 7-day culture were collected by centrifugation, re-suspended in 1 ml TRIzol® Reagent (Invitrogen 15,596,026), frozen at −80°C and used as the reference transcriptome representing bacteria prior to challenge of the larvae. For day 1 and day postinfection, we used groups of 30 larvae, and for day 3 and day 4 postinfection and control (uninfected), we used groups of 10 larvae for RNA extraction. At each time larvae were placed on ice for 5 min, hemolymph isolated and hemocytes collected by centrifugation, re-suspended in 1 ml TRIzol® Reagent and frozen at −80°C. The entire experiment was repeated on two more occasions.

### Bacterial RNA extraction and RNA sequencing (RNASeq) for transcriptional studies

Total eukaryotic and prokaryotic RNA was extracted using a Direct-zol™ RNA MiniPrep Kit (Zymo Research R2052). Contaminating DNA was removed by DNAse I treatment (Ambion AM2222) which was confirmed by PCR using the 16S-F (5ʹ TTCGGACCTCGTGCTATAAG 3ʹ) and 16S-R (5ʹ ACTACCAGGGTCTCTAATCC 3ʹ) primer pair. Presence of *C. burnetii* mRNA in the DNA-free total RNA extract was confirmed by reverse transcriptase PCR (Qiagen OneStep RT-PCR Kit 210212) using the *com1*-F (5` GACAGAAGCGCAACAAGAAG 3`) and *com1*-R (5ʹ ATAATTGGCCGTCGACACTG 3ʹ), and the *rpoB*-F (5ʹ TACCAGCTATTCTGGGTACG 3ʹ) and *rpoB*-R (5ʹ CAACCACGAACCACGATAAG 3ʹ) primer pairs. No amplification occurred in the PBS or uninfected control samples. The RNA integrity number (RIN) score and RNA concentration was determined using an Agilent 4200 TapeStation System. The RIN score was 9.3 or over for all extracts, indicating minimal RNA degradation. Five micrograms of RNA isolated from infected larvae, and 2 μg RNA isolated from bacterial culture (both in triplicate) was further processed. Eukaryotic mRNA was depleted using TruSeq mRNA stranded (oligo-dT) beads (TruSeq RNA Library Preparation Kit v2, Illumina RS-122-2001). The bacterial mRNA was then enriched using the riboPOOL Human:Pan-prokaryote or the riboPOOL Pan-prokaryote kits (Source Bioscience rdS0501 or rdCS0501, respectively). RNASeq libraries were prepared using the Illumina TruSeq Stranded mRNA Library Prep Kit according to the manufacturer’s protocol. The concentration, quality, and integrity of the libraries were analyzed using the Agilent 4200 TapeStation System. Sequencing was performed at the University of Exeter Sequencing Facility using an Illumina HiSeq System benchtop sequencing instrument (read length: 125 bp, read type: paired end). Sequence data are available at the National Center for Biotechnology Information, Sequence Read Archive under accession PRJNA611927. Reads from RNASeq were mapped to the *C. burnetii* strain RSA493 genome (GCA_000007765.2_ASM776v2) using Tophat [29]. Cufflinks [30] was used for transcript assembly of individual samples. All assemblies were merged to create a reference transcript, which was used to quantify transcript expression using Salmon [31]. DESeq [32] was then used to find differentially expressed genes. Transcripts with a *p*-value <0.05 and more than twofold differential expression were considered significantly expressed.

### Reverse transcription quantitative PCR (RT-qPCR) for validation of the RNASeq data

1 μg of bacterial RNA was reverse transcribed to generate cDNA using random hexamers and the Superscript III Reverse Transcriptase Synthesis System (Invitrogen 18080051) according to the manufacturer’s recommendations. qPCR was performed with primers annealing to internal regions of the target genes (Supplementary Table S1) using SYBR™ Green PCR Master Mix (Applied Biosystems 4309155) on a QuantStudio 6 Flex Real-Time PCR System (Applied Biosystems). Data was obtained through QuantStudio Real-Time PCR Software v1.3. Relative mRNA abundances were

$$Ratio=\frac{Effciency{\left(target\right)}^{CT(target,\;untreated)-CT(target,\;treated)}}{Effciency{\left(ref\right)}^{CT(ref,\;untreated)-CT(ref,\;treated)}}$$

calculated by the following equation using the 16S rRNA gene [33] to normalize the results:

### Bioinformatic and statistic tools

Cellular localization of the predicted proteins encoded in the *C. burnetii* genome was predicted using PSORTb 3.0 [34]. Functional analyses of *C. burnetii* genes were performed using the Cluster of Orthologous Gene classification (COG) [35]. Regression analysis was performed using GraphPad Prism Version 5.03.

## Results and discussion

### *Infection of* G. mellonella *larvae with* C. burnetii *NMII*

**Figure 1.**
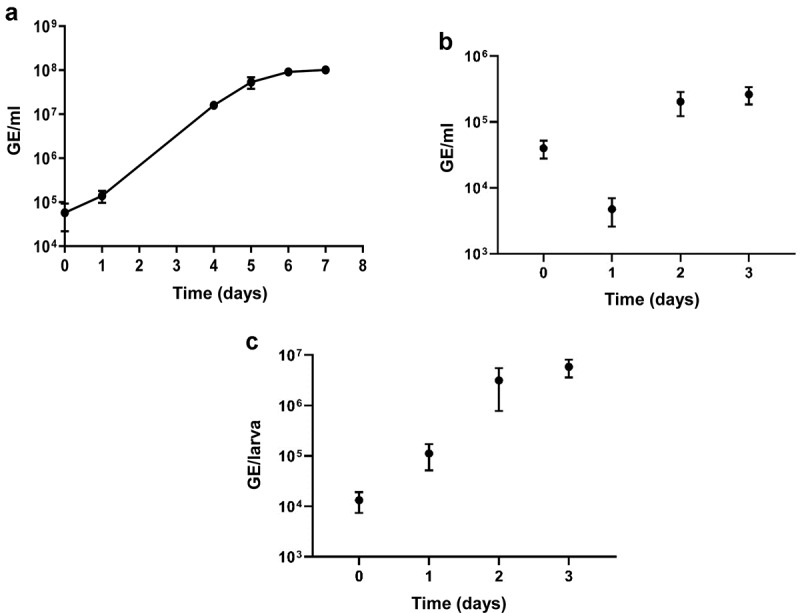
Expansion of *C. burnetii* NMII in ACCM-2 medium or in *G. mellonella* larvae. Larvae were injected with 10^6^ GE of bacteria and bacteria were enumerated by qPCR at times indicated. (a) number of bacteria in ACCM-2; mean and SEM of triplicates (b) number of bacteria in *G. mellonella* hemocytes; mean and SEM from 3 larvae (c) number of bacteria in *G. mellonella* whole larvae homogenates; mean and SEM from 6 larvae.

*G. mellonella* larvae were challenged with *C. burnetii* NMII as described previously [21]. The growth rate of the NMII strain in *G. mellonella* larvae (mean generation time 5 hours) was faster than the growth rate in ACCM-2 medium (mean generation time 10.6 hours) (Figure 1). Whilst we saw a steady increase in *C. burnetii* burden in *G. mellonella* larvae over the course of the infection (Figure 1(c)), previously Selim et al., using the NMI strain, reported [26] a decline in *C. burnetii* burden for up to 48 hours postchallenge, followed by an increase in burden at 72 hours and up to 168 hours postchallenge. These differences might reflect differences in the challenge strain or in the source of the *G. mellonella* larvae used. When hemocytes were isolated from larvae immediately after challenge (T0) we found that most of the bacteria were hemocyte associated (Figure 1(b)). There was a subsequent decline in number of bacteria at 1 day postinfection which may be due to uptake and killing of bacteria before a phase of bacterial growth.

**Figure 2.**
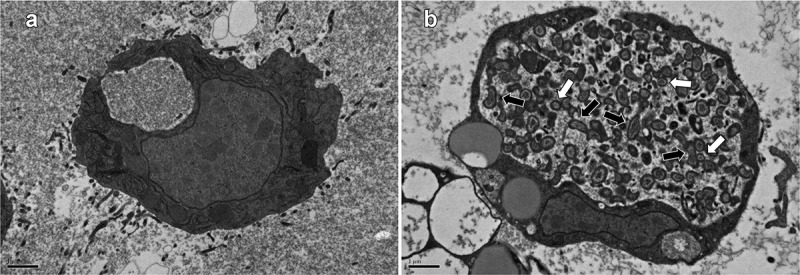
Transmission electron microscopy of *G. mellonella* hemocytes postinfection with *C. burnetii. G. mellonella* larvae were injected with *C. burnetii* NMII at a dose of 10^6^ GE/larvae and at 3 days postinfection larvae were bled, and hemocytes visualized by a transmission electron microscope. (a) Uninfected controls with no bacteria being visible. (b) Hemocytes from infected larvae with a *Coxiella*-containing vacuole clearly visible, which spread to fill the entire cell cytoplasm. Arrows indicate proposed LCVs (black) and SCVs (white). Images shown are representative of 50 control images and 50 images of infected hemocytes. Scale bar = 1 μm.

Next, TEM was used to image hemocytes from larvae at 3 days postinfection with the NMII strain. This revealed bacteria in clearly defined vacuoles which had filled the entire hemocyte cytoplasm (Figure 2(b)). These vacuoles appeared to contain *C. burnetii* in both the SCV (small cell variant) and LCV (large cell variant) forms, and the vacuoles appeared to be morphologically similar to the *Coxiella*-containing vacuoles previously reported in macrophages infected with *C. burnetii* [5].

We next extracted bacterial mRNA from the hemocytes isolated from *G. mellonella* larvae that had been infected with *C. burnetii* NMII. The RIN score of the isolated RNA was at least 9.3, indicating minimal degradation. We measured the mRNA levels of two housekeeping genes, *com1* and *rpoB*, by RT-qPCR using total RNA (mixture of eukaryotic and prokaryotic total RNA) as template extracted from the hemocytes of infected *G. mellonella* larvae. The levels of *com1* and *rpoB* mRNA increased as the infection progressed (Supplementary Figure S1), which corroborates the earlier finding that the numbers of *C. burnetii* cells within the hemocytes of *G. mellonella* increased after inoculation of the larvae (Figure 1(b)). This increase might be a combination of intracellular growth and increasing uptake of bacteria by hemocytes.

### *Mapping the transcriptome of* C. burnetii *by RNASeq*

To investigate how *C. burnetii* adapts to infection of *G. mellonella* larvae, the transcriptional landscapes of bacteria were profiled, using RNASeq, at 1, 2, 3, and 4 days postinfection. We used three groups each of 10–30 larvae for each time point analyzed and excluded any larvae that had died. We compared these profiles to the transcriptional profile of the challenge, i.e. bacteria grown in ACCM-2 liquid medium for 7 days.

First, we carried out principal component analysis (PCA) to compare the datasets we obtained from three biological replicates. This showed that sample replicates clustered together, indicating experimental reproducibility (Supplementary Figure S2). Next, we determined the number of genes expressed. We detected broadly similar numbers of genes expressed *in vitro* (n = 1780) or in bacteria isolated from *G. mellonella* at the four time points (n = 1708–1727). Many of the genes (n = 1668) were expressed in all of the samples, but some were expressed only at different stages of infection or only during *in vivo* or *in vitro* growth (Supplementary Table S2).

We investigated how similar the global transcriptomes were to each other using regression analysis (Supplementary Figure S3). We found that the *in vitro* bacterial transcriptome prior to injection was more similar to that in *G. mellonella* at the later stages of the infection (at 3 day and 4 days postinfection) than to that at earlier time points (1 days and 2 days postinfection). This is confirmed by the distance of the *in vivo* time point from the *in vitro* time points on the first principle component (accounting for 77% of the variance, Supplementary Figure S2), as well as in a heat map of the expression data (Supplementary Figure S4).

In addition, we measured changes in gene expression. Compared to *in vitro* grown bacteria prior to challenge, we found that 467, 386, 370, or 320 genes were significantly upregulated at 1, 2, 3, or 4 days postinfection, respectively. On the other hand, 481, 397, 399, or 394 genes were significantly downregulated at 1, 2, 3, or 4 days postinfection, respectively (Supplementary Table S3). The numbers of significantly upregulated genes, as well as the mean fold change in their expression (8.5-, 7.4-, 6.9-, or 6-fold at 1, 2, 3, or 4 days postinfection, respectively) was greater at earlier times postinfection. Overall, our findings suggest that the transcriptome of bacteria at 3 and 4 days postinfection are more similar to the conditions in ACCM-2 medium than the transcriptomes of bacteria at days 1 or 2 postinfection are.

Nineteen genes were selected to validate the RNASeq expression data, using RT-qPCR with 16S rRNA as an internal control [33] (Supplementary Figure S5). We obtained similar results for fold change difference in the expression of the selected genes by RT-qPCR and RNASeq. Overall, we concluded that the RNASeq data provided a robust picture of the transcriptome of *C. burnetii*.

### *Cell variants present in the hemocytes of* G. mellonella

*C. burnetii* undergoes a biphasic developmental cycle during infection of eukaryotic host cells, characterized by the transition of metabolically dormant, non-replicating SCV into metabolically active and replicating LCV [36]. This transition also occurs during growth in ACCM-2 medium [37]. Previous studies have identified genes that are associated with the LCV or SCV forms of *C. burnetii* [37–39]. Based on these we created lists of LCV-associated or SCV-associated genes (Supplementary Table S4).

Compared to the challenge (bacteria grown in ACCM-2 medium for 7 days and therefore representing a mixture of LCV and SCV cells), 163 of the 325 LCV-associated genes (50%) were significantly upregulated at one or more time points during infection of *G. mellonella* (Supplementary Figure S6.A). Conversely, of 197 SCV-associated genes, the expression of 141 (72%) were significantly downregulated at least at one time point during infection (Supplementary Figure S6.B). The numbers of the LCV- and SCV-associated genes that were significantly upregulated or significantly downregulated at each time point in *G. mellonella* are shown in Supplementary Table S5. These findings suggest a progressive decrease in the number of LCV cells and in parallel a progressive increase in the number of SCV cells, over 4 days of infection in *G. mellonella*, even though relative abundances of each cell variant cannot be extrapolated from our data. Nevertheless, our TEM images (see Figure 2(b)) clearly show that SCV cells are present in *Galleria* hemocytes at day 3 postinfection, thereby corroborating that the *Galleria* infection model allows both cell variants to exist.

### *Genes potentially important during infection of* G. mellonella

Genes that are upregulated *in vivo* may be important for infection. In a previous study with *Burkholderia thailandensis*, we showed that during infection of macrophages 20 of the 25 most highly upregulated genes encoded virulence factors [40]. We identified the 25 most highly upregulated genes in *C. burnetii* at 1, 2, 3, or 4 days postinfection of *G. mellonella*, and combining these lists identified 46 genes upregulated at one or more time points (Supplementary Table S6). Of these, 30 (65%) genes encoded hypothetical proteins. Transposon mutants in two of these genes, CBU_1716b and CBU_2003a, were found to either show defects in intracellular replication and a cytotoxic phenotype in cell culture, or a defect in internalization, respectively [41]. Transposon mutants in three other genes encoding hypothetical proteins CBU_0006a, CBU_0008, and CBU_0037a showed no defects in cell culture [13,15], whereas experimental data was lacking on the remaining hypothetical proteins. These hypothetical proteins may serve as targets for future studies on the virulence of *C. burnetii*.

Of the remaining 16 genes, 7 encoded proteins involved in translation. This finding is in accordance with the faster growth of *C. burnetii* in *G. mellonella* compared to growth in ACCM-2 medium (Figure 1), and the greater demand for protein synthesis. CBU_0676, CBU_0677, and CBU_0678 are predicted to be involved in LPS biosynthesis [42,43], particularly the biosynthesis of virenose, one of two sugars unique to the O-antigen of *C. burnetii* [44]. However, since these three ORFs are located directly upstream the deletion responsible for the rough LPS phenotype in NMII [45], we cannot exclude that the up-regulation of these genes is a result of a polar effect of this large deletion. CBU_1004 (*bioC.2*), CBU_1006 (*bioF*), CBU_1007 (*bioB*), and CBU_1008 (*bioA*) are predicted to be involved in biotin synthesis. Biotin has been previously associated with the virulence of *C. burnetii* [46] and with virulence of other pathogens such as *Francisella tularensis* and *Mycobacterium tuberculosis* [47,48]. Finally, CBU_1477 (*ahpC*) and CBU_1478 (*ahpD*) encode a peroxiredoxin (ROS scavenging enzyme) and a peroxiredoxin reductase, respectively. These proteins have previously been identified as putative virulence factors of *C. burnetii* [10,49,50] and may play roles in SCV formation [37]. Finally, CBU_2007 encodes a putative T4SS effector [16]. However, transposon insertions in this gene did not result in any defects in replication or CCV formation in cell culture [13,18].

### Type IV secretion system (T4SS)

One of the major virulence factors of *C. burnetii* is the Dot/Icm type 4 secretion system (T4SS) [51]. The T4SS is essential for effector protein secretion, the trafficking of bacteria in host cells, and replication within macrophages. Therefore, we investigated the expression of genes encoding T4SS core components and effectors in more detail.

The genes encoding the structural (core) components of the T4SS were expressed but were not significantly upregulated at any time in *G. mellonella*, compared to expression in bacteria grown in ACCM-2 medium (Supplementary Table S7). Previous studies have shown that the T4SS core components are not upregulated during infection of buffalo green monkey (BGM) cells or mice [50]. However, in Vero cells, expression of the *icmX, icmW, icmV, dotA, dotB*, and *icmT* genes increased during the first 24 hours of infection, but subsequently decreased [52]. Therefore, it is possible that upregulation of the T4SS core components of *C. burnetii* occurred during the first 24 hours of infection of *G. mellonella* and before we isolated bacterial mRNA. Also, ACCM-2 mimics the conditions in the acidified endosome [20], where the T4SS is functional, and we found that transcripts of many core components were abundant during *in vitro* growth. Overall, our findings are consistent with previous work showing that the T4SS system plays a role in infection of *G. mellonella* [21,27].

**Figure 3.**
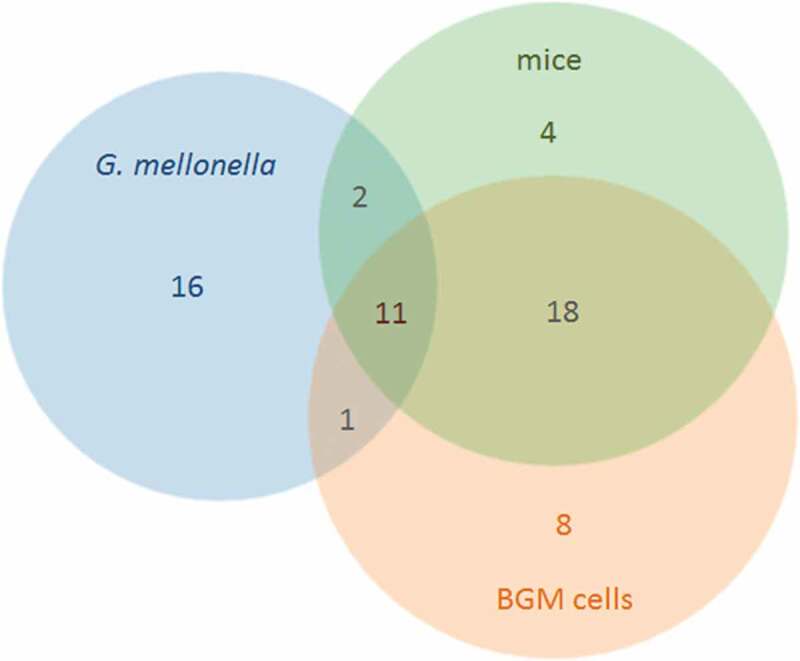
Significantly upregulated T4SS effectors in *G. mellonella*, in BGM cells and in mice. Datasets compared included the RNASeq results described in this study, as well as microarray data of infected BGM cells and mice performed by Kuley *et al*. 2015 [50], as described in Supplementary Table S7.

We found that 30 T4SS effectors were significantly upregulated at least at one time point during infection of *G. mellonella* (Figure 3 and Supplementary Table S7). Of these, nine were significantly upregulated over the course of the infection, and two of these (CBU_1685 and CBU_1863) have previously been shown to be significantly upregulated after infection of mice [50]. Eleven effectors upregulated in *G. mellonella* have as well been shown to be upregulated after infection of BGM cells and mice [50]. Effectors that are upregulated in BGM cells, in mice, and in *G. mellonella* might have general roles during infection. We found seven effectors that were upregulated in *G. mellonella* and mice but not in BGM cells. Differences in the expression of T4SS effectors in different hosts (Figure 3 and Supplementary Table S7) suggests that different but overlapping sets of effectors are required in different hosts. This suggestion is supported by the observation that different repertoires of T4SS effectors are encoded in the genomes of strains isolated from different niches [11,16,18,53].

### *Transcriptomic profiles of* C. burnetii *during infection of different hosts*

We compared the significantly regulated genes of *C. burnetii* during infection of BGM cells, during chronic infection of mice [50] and during infection of *G. mellonella* (Supplementary Figure S7, Supplementary Table S8, and Supplementary Table S9). Regression analysis reveals that the changes in the transcriptome during infection of *G. mellonella* according to our RNASeq data are more similar to the transcriptome changes in BGM cells than to the transcriptome changes in mice (Table S9). This may reflect differences in the growth rates in these different hosts, the acute nature of the infection in *G. mellonella* and our focus on the infection of hemocytes.

We identified a total of 253 genes that are significantly upregulated in *G. mellonella* at all time points (at 1, 2, 3 and 4 days post-infection) compared to growth in ACCM-2 broth (Supplementary Table S3). Of these, 49 are also significantly upregulated after infection of BGM cells and mice (Table 1), suggesting that these genes might be important in infection of all three hosts. These commonly upregulated genes included CBU_1477 (*ahpC*) and CBU_1478 (*ahpD*), the CBU_1003-CBU_1008 operon (*bioA, bioB, bioC.2, bioF,* and *bioH* genes), CBU_0676, CBU_0677, CBU_0844, CBU_0846 (*ugd*) and CBU_1655 involved in LPS/virenose synthesis [42,43,54] and genes involved in translation (CBU_0569, CBU_1430, CBU_1474, CBU_1817, and CBU_1842). Of these, CBU_0844 has homology to CBU_0677, and CBU_1655 has homology to the CBU_0678 gene. These genes and their products are discussed in more detail above.

**Table 1.** Genes commonly upregulated in *G. mellonella*, BGM cells, and mice. Light shaded cells show genes that were 2- to 10-fold upregulated and dark shaded cells show genes that were more than 10-fold upregulated. In all cases, gene expression was compared to bacteria grown in ACCM-2 medium for 7 days (*G. mellonella* data) or grown to stationary phase in ACCM-2 (BGM cells or mice data). The BGM and mice data is adapted from Kuley *et al.* [50]. Detailed expression data for these genes are found in Supplementary Table S3.

Locus tag	Gene symbol	Encoded protein	*G. mellonella*				BGM cells	mice
			1d	2d	3d	4d		
CBU_0037a		hypothetical protein						
CBU_0074		hypothetical protein						
CBU_0312	*folD*	methylenetetrahydrofolate dehydrogenase (NADP+)						
CBU_0387	*cgtA*	GTP-binding protein (probably involved in DNA repair)						
CBU_0557	*holA*	DNA polymerase III delta subunit						
CBU_0558		rare lipoprotein B precursor						
CBU_0569		tRNA 2-methylthioadenosine synthase						
CBU_0607	*mvaD*	diphosphomevalonate decarboxylase						
CBU_0608		phosphomevalonate kinase						
CBU_0609		mevalonate kinase						
CBU_0627		hypothetical protein						
CBU_0672		hypothetical protein						
CBU_0676		UDP-glucose 4-epimerase						
CBU_0677		NAD dependent epimerase/dehydratase family						
CBU_0844		UDP-N-acetylglucosamine 4-epimerase						
CBU_0846	*ugd*	UDP-glucose 6-dehydrogenase						
CBU_1004	*bioC.2*	biotin synthesis protein						
CBU_1005	*bioH*	carboxylesterase						
CBU_1006	*bioF*	8-amino-7-oxononanoate synthase						
CBU_1007	*bioB*	biotin synthase						
CBU_1008	*bioA*	adenosylmethionine-8-amino-7-oxononanoate aminotransferase						
CBU_1010		hypothetical protein						
CBU_1117	*etfA*	electron transfer flavoprotein alpha-subunit						
CBU_1141	*secF*	protein translocase subunit						
CBU_1181	*thiI*	thiamine biosynthesis protein						
CBU_1273		pyrophosphate–fructose 6-phosphate 1-phosphotransferase						
CBU_1281	*carB*	carbamoyl-phosphate synthase large chain						
CBU_1285		multidrug resistance protein B						
CBU_1289	*dnaJ*	chaperone protein						
CBU_1323a		hypothetical cytosolic protein						
CBU_1430	*truB*	tRNA pseudouridine synthase B						
CBU_1435	*nuoN*	NADH-quinone oxidoreductase chain N						
CBU_1474	*gatA*	aspartyl/glutamyl-tRNA(Asn/Gln) amidotransferase subunit A						
CBU_1477	*ahpC*	peroxiredoxin						
CBU_1478	*ahpD*	peroxiredoxin reductase (NAD(P)H)						
CBU_1580		ATPase						
CBU_1655	*rfaE*	D-glycero-D-manno-heptose-7-phosphate 1-kinase						
CBU_1685		hypothetical protein						
CBU_1696	*rnfB*	electron transport complex protein						
CBU_1697	*Nth*	endonuclease III						
CBU_1721		hypothetical protein						
CBU_1726	*accC*	biotin carboxylase						
CBU_1777		hypothetical protein						
CBU_1817		lysyl-tRNA synthetase						
CBU_1839		aminobutyraldehyde dehydrogenase						
CBU_1842		GTP-binding protein probable translation factor						
CBU_1920	*yidC*	60 kDa inner membrane protein						
CBU_1957	*pntB*	NAD(P) transhydrogenase subunit beta						
CBU_2012	*hslU*	ATP-dependent endopeptidase hsl ATP-binding subunit						

There may also be a requirement for folic acid and thiamine in all three hosts, since the *folD* (CBU_0312) and *thiI* (CBU_1181) genes were upregulated in all *G. mellonella*, mice, and BGM cells. Genes encoding components of the mevalonate pathway (CBU_0607, CBU_0608, and CBU_0609), which has a role in the synthesis of isoprenoids, were also significantly upregulated during infection of all three hosts. CBU_0037a has also been associated with lipid metabolism. Isoprenoids are a class of lipids that have a wide variety of roles in physiological processes in bacteria, such as survival or host–pathogen interactions. In *Listeria monocytogenes, M. tuberculosis*, and *Klebsiella pneumoniae*, mutants of the mevalonate pathway show increased virulence in mice [55–59]. Given that these molecules have vital functions, isoprenoid biosynthetic enzymes are considered as potential drug targets against bacterial pathogens [60]. Finally, a T4SS effector, CBU_1685 was significantly upregulated in all hosts, suggesting general importance of CBU_1685 in interfering with host defense mechanisms and possibly in bacterial survival. Beare *et al*. [61] experimentally confirmed (by luciferase gene reporter assay) that CBU_1685 is a T4SS effector.

In summary, the findings we report here provide important new insights into the utility of the *G. mellonella* infection model to study virulence of *C. burnetii*. Our findings show that many features of the infection reflect features of the infection in other hosts such as cultured cells and mice. Whilst we cannot exclude the possibility that *C. burnetii* replicates in a range of cell types in *G. mellonella*, our findings indicate that the bacteria replicate in hemocytes, and occupy a vacuole which appears to be similar to the CCV seen in infected mammalian phagocytes. In mammalian cells, the formation of this vacuole, which becomes phagolysosome-like, is directed by *C. burnetii* [62]. Our work reveals a wide range of genes that are upregulated during infection of *G. mellonella*. Many of these genes encode proteins whose function is not known, and these should be targets for future studies on the virulence of *C. burnetii*.

We found many similarities in the gene expression profiles of bacteria from hemocytes, BGM cells, and mice, and not unexpectedly we also found many differences. These differences likely reflect the different features of these different hosts. Whilst there are similarities between the *G. mellonella* and the mouse infection model, it is important to note that in *G. mellonella* the NMII strain of *C. burnetii* behaves very much like the NMI strain. In contrast, the NMII strain is highly attenuated in mice. Therefore, it appears that the *G. mellonella* infection model can be used for meaningful studies on virulence mechanisms of *C. burnetii* with a strain that poses a lower risk of infection of humans.

## Supplementary Material

Supplemental Material
