# Supplementary material for: Coxiella burnetii replicates in Galleria mellonella hemocytes and transcriptome mapping reveals in vivo regulated genes
Source: Virulence. 2020 Sep 24;11(1):1268–78. doi: 10.1080/21505594.2020.1819111 (PMC7549970; doi:10.1080/21505594.2020.1819111)
Supplement: Supplemental Material [file KVIR_A_1819111_SM6611.zip › Supplementary Figures_v4.docx]

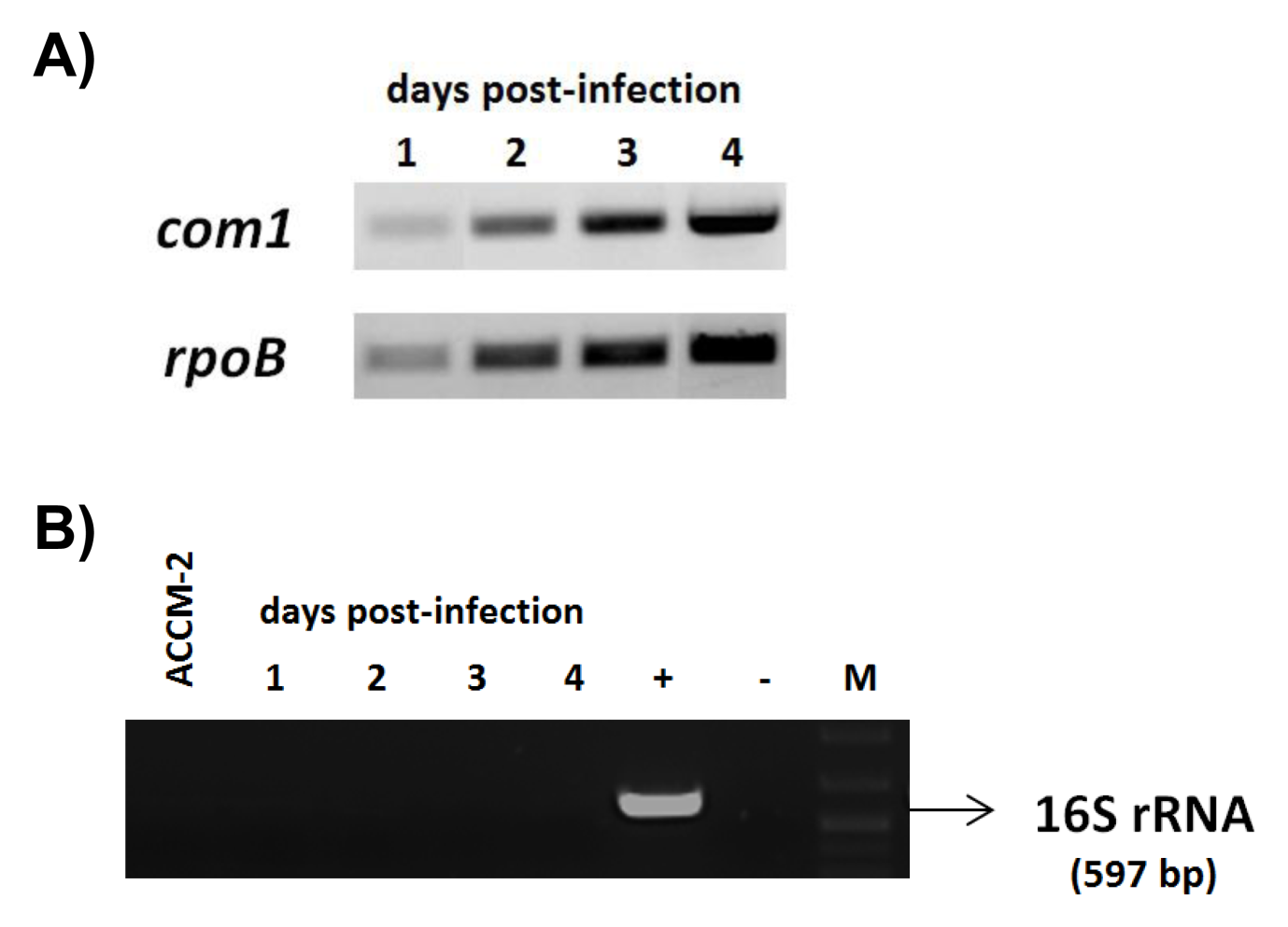


**Supplementary Figure S1.** Quantification of *Coxiella*-specific RNA in total RNA extracted from infected *G. mellonella* haemocytes. (A) RNA was extracted from infected larvae at the times indicated (haemocytes isolated and pooled from 10 larvae) and used to amplify the *com1* and *rpoB* housekeeping genes of *C. burnetii* ^33^ by RT-PCR. Equal volumes of RT-PCR products were subjected to gel electrophoresis and gels were imaged using a Chemidoc system (Bio-Rad). (B) Confirmation of DNAse I treatment of RNA samples by PCR prior to RT-PCR. Purified RNA was subjected to DNAse I treatment and the 16S rRNA gene was amplified by PCR. The results showed that the DNAse I treatment was successful as no band appeared in the RNA samples. ACCM-2: *C. burnetii* grown *in vitro*, +: DNA positive control, -: water negative control, M: DNA molecular weight marker.


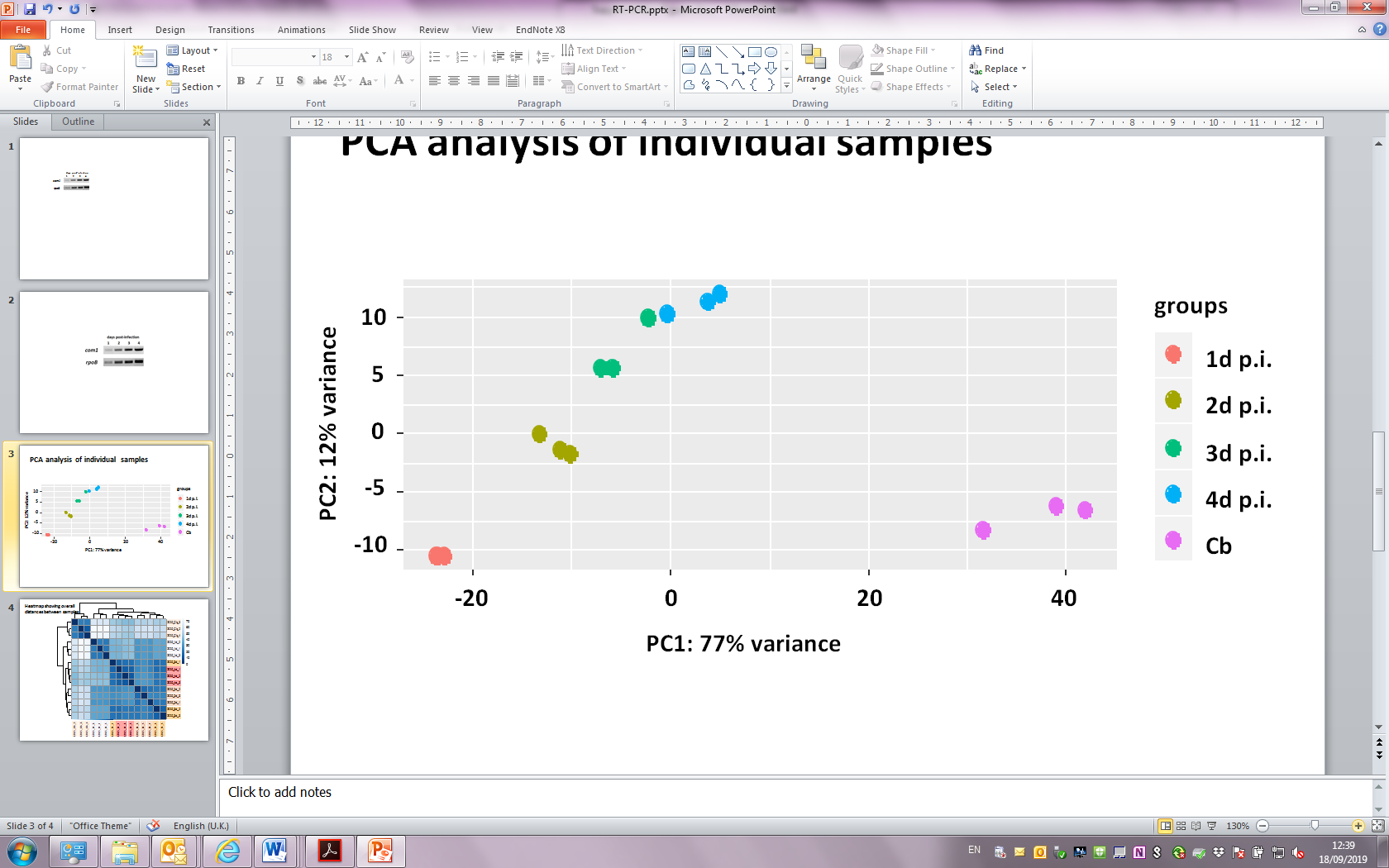


**Supplementary Figure S2.** Principal component analysis on global expression profiles of *C. burnetii* NMII obtained from bacteria grown *in vitro* (“Cb”) and during *G. mellonella* infection (Days 1-4 post infection). Replicates of most of the samples are tightly clustered indicating experimental reproducibility.


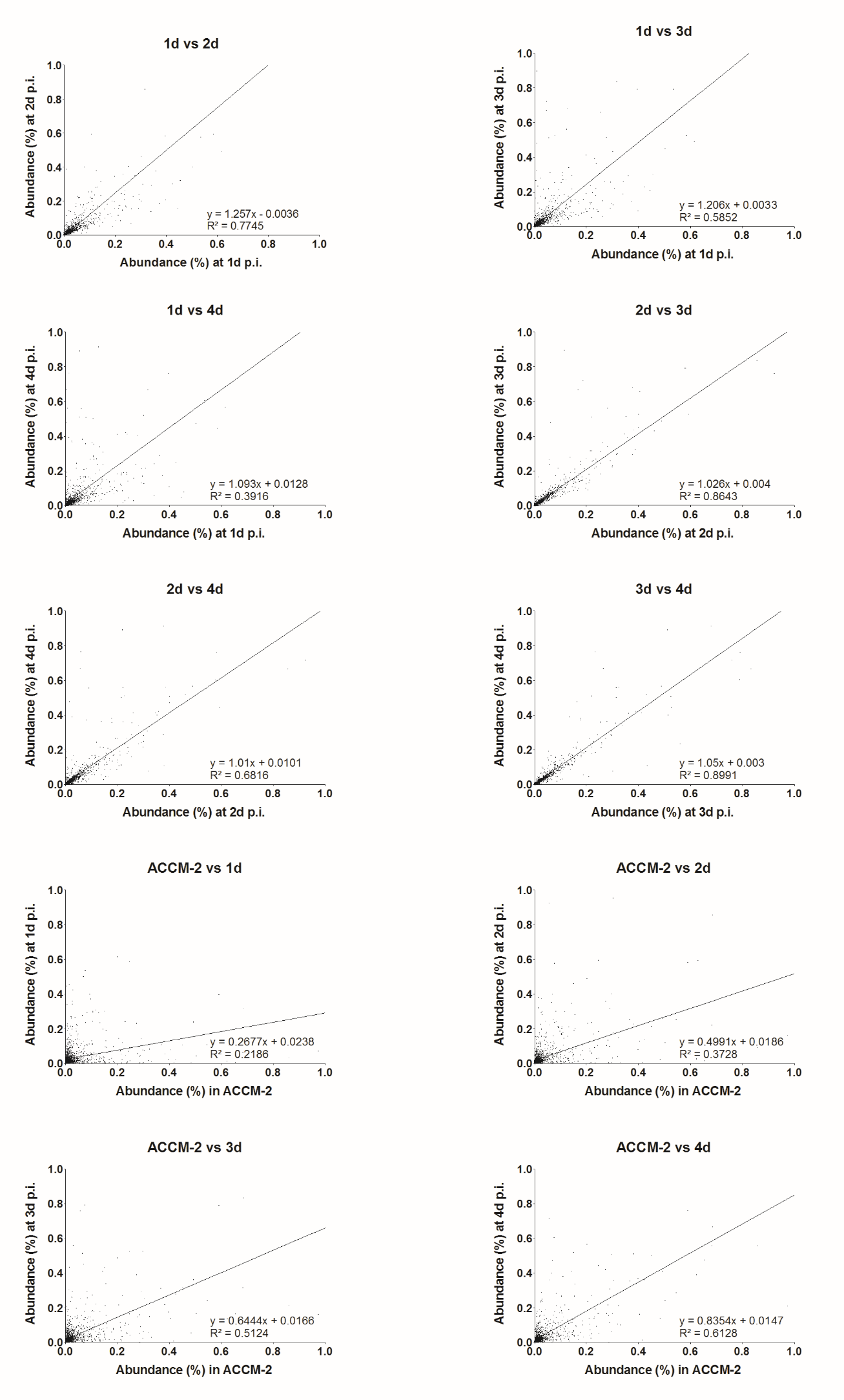


**Supplementary Figure S3.** Scatter plots of transcript abundances in *C. burnetii* grown *in vitro* or during *G. mellonella* infection. Pairwise plots of normalised abundances of transcripts are shown. Data points above 1% abundance were included in our analysis but are not shown on the graphs.. Data points of genes with an abundance of >10% and which also showed a highly skewed difference in expression between the compared datasets were excluded from analysis (CBU_0110a excluded from all panels except ACCM-2 vs 4d, and CBU_1267a from panels ACCM-2 vs 1d, 2d, 3d and 4d). The equation and R square (R^2^) of the linear regression line (black line) are included in each plot.


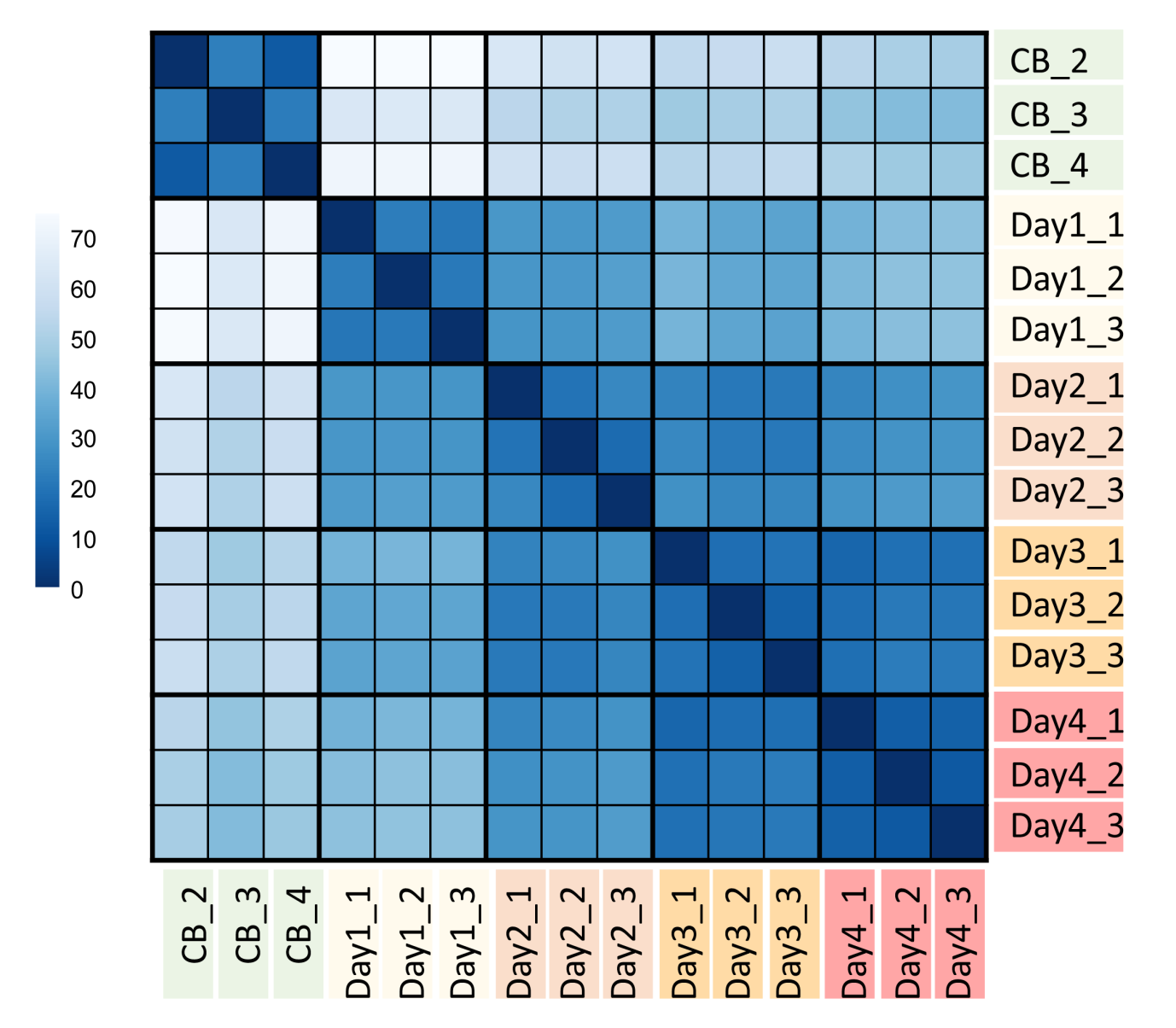


**Supplementary Figure S4.** Heatmap showing overall distances between different samples on global gene expression profiles of *C. burnetii* NMII obtained from bacteria grown *in vitro* (“CB”) and during *G. mellonella* infection (Day 1 – Day 4 post infection).


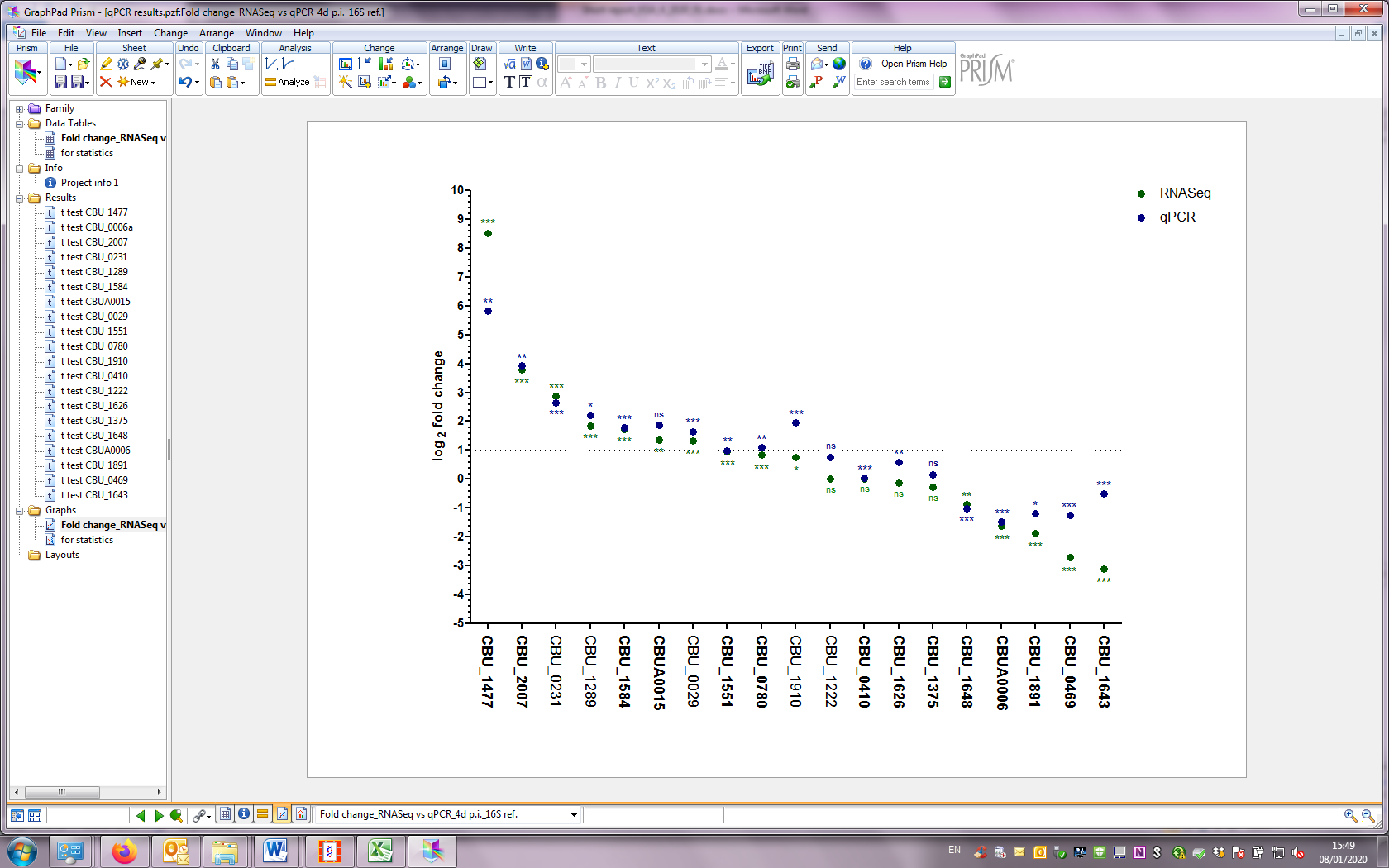


**Supplementary Figure S5. Validation of RNASeq data by RT-qPCR.** RNASeq (green) and RT-qPCR (blue) data at 4-day post-infection of *G. mellonella* compared to *in vitro* growth in ACCM-2 medium. Tested genes include putative virulence-related factors (shown in bold fonts), and significantly and non-significantly regulated genes. 16S rRNA was used as internal control in the RT-PCR. Dotted lines show 2-fold change in expression (cut-off for significance). Asterisks on the graph indicate statistical significance (*p<0.05, **p<0.01, ***p<0.001).


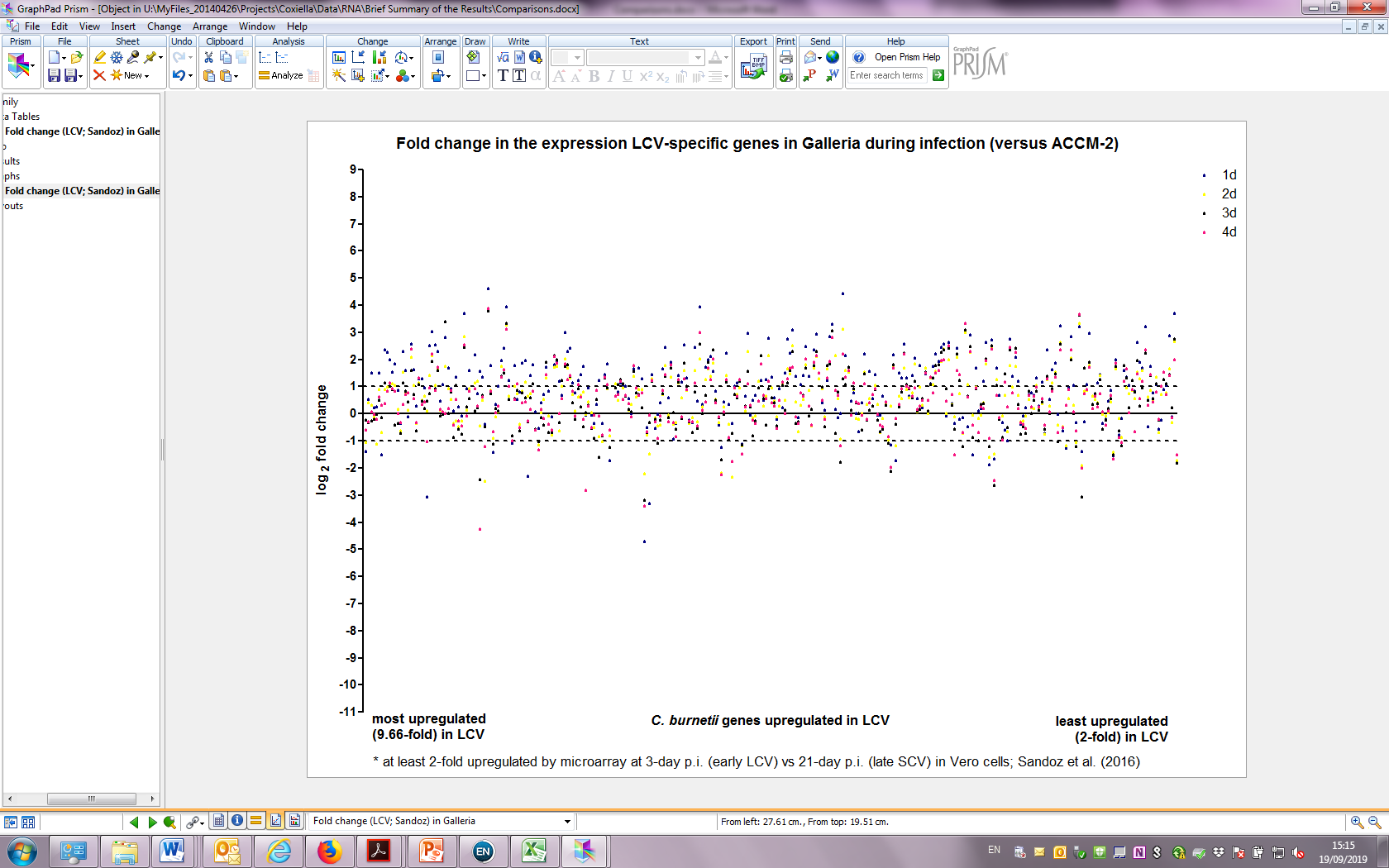


**A**

**B**


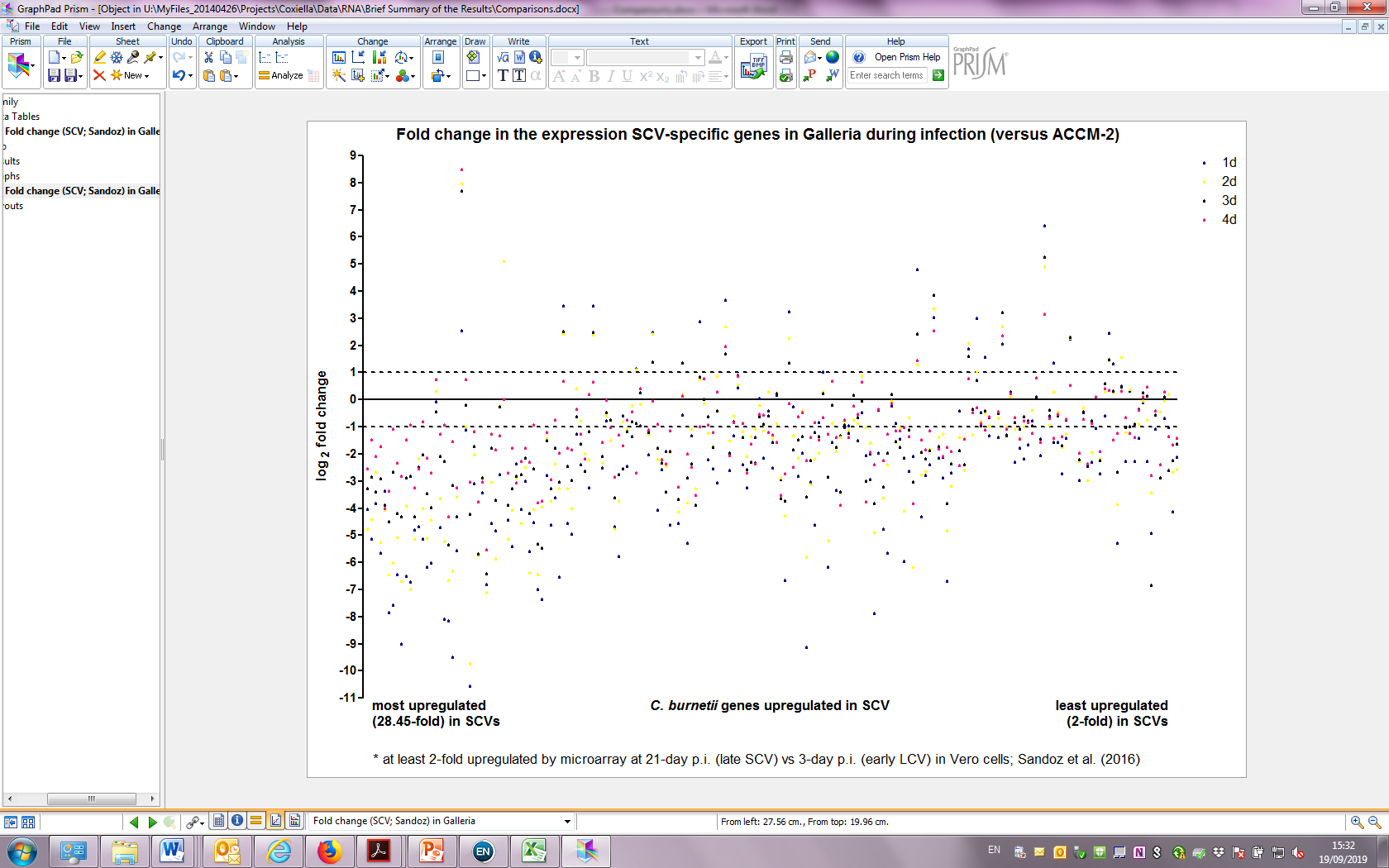


**Supplementary Figure S6.** Fold change difference in the expression of LCV- and SCV-specific genes during infection of *G. mellonella* compared to *in vitro* growth. Genes were selected based on their expression during infection of Vero cells as analysed by microarray by Sandoz *et al*. 2016 ^38^. (A) LCV-specific genes (*n* = 325). Genes significantly upregulated at 3-day post-infection (early LCV) compared to 21-day post-infection (late SCV) in Vero cells were considered to be important in LCV and ranked according to their level of upregulation along the X-axis. (B) SCV-specific genes (*n* = 197). Genes significantly upregulated at 21-day post-infection (late SCV) compared to 3-day post-infection (early LCV) in Vero cells were considered to be important in SCV and ranked according to their level of upregulation along the X-axis.

**A**

**B**

**[Supplementary Figure S7_part1]**

**C**

**D**

**[Supplementary Figure S7_part2]**

**E**

**F**

**[Supplementary Figure S7_part3]**

**G**

**H**

**Supplementary Figure S7.** Comparison of significantly regulated *C. burnetii* genes in *G. mellonella*, BGM cells and mice. Quartet graphs of *G. mellonella* infection were created against infection of BGM cells (A-D ^51^) and infection of mice (E-H ^51^) and colour coded: orange circles = significantly regulated in both *G. mellonella* and BGM cells (A-D) or mice (E-H); black circles non-significantly regulated in *G. mellonella* but significantly regulated in BGM cells (A-D) or mice (E-H). The numbers of common significantly regulated genes in each comparison are shown Supplementary Table S8.
