# Supplementary material for: Coxiella burnetii replicates in Galleria mellonella hemocytes and transcriptome mapping reveals in vivo regulated genes
Source: Virulence. 2020 Sep 24;11(1):1268–78. doi: 10.1080/21505594.2020.1819111 (PMC7549970; doi:10.1080/21505594.2020.1819111)
Supplement: Supplemental Material [file KVIR_A_1819111_SM6611.zip › Supplementary Table S5_v3.docx]

**Supplementary Table S5.** Numbers of significantly regulated LCV-associated or SCV-associated genes in *G. mellonella* at 1, 2, 3 and 4 days post-infection.

|  |  | **1-day p.i.** | **2-day p.i.** | **3-day p.i.** | **4-day p.i.** | **Total**  **(1-4 days p.i.)** |
| --- | --- | --- | --- | --- | --- | --- |
| **LCV-associated genes (325)** | **Significantly upregulated** | 146 | 103 | 93 | 87 | 163 |
|  | **Significantly downregulated** | 22 | 20 | 21 | 13 | 33 |
| **SCV-associated genes (197)** | **Significantly upregulated** | 11 | 10 | 8 | 7 | 14 |
|  | **Significantly downregulated** | 131 | 113 | 109 | 103 | 141 |
