# Supplementary material for: Coxiella burnetii replicates in Galleria mellonella hemocytes and transcriptome mapping reveals in vivo regulated genes
Source: Virulence. 2020 Sep 24;11(1):1268–78. doi: 10.1080/21505594.2020.1819111 (PMC7549970; doi:10.1080/21505594.2020.1819111)
Supplement: Supplemental Material [file KVIR_A_1819111_SM6611.zip › Supplementary Table S6_v2.docx]

**Supplementary Table S6.** *C. burnetii* genes potentially important during infection of *G. mellonella*. Detailed expression data for these genes are shown in Supplementary Table S3.

| **Locus tag** | **Gene symbol** | **Name** |
| --- | --- | --- |
| **CBU_0006a** |  | hypothetical protein |
| **CBU_0008** |  | hypothetical protein |
| **CBU_0037a** |  | hypothetical protein |
| **CBU_0074** |  | hypothetical protein |
| **CBU_0110a** |  | hypothetical protein |
| **CBU_0221b** |  | protein translation elongation factor Tu (EF-TU) |
| **CBU_0226** | *rplK* | LSU ribosomal protein L11P |
| **CBU_0227** | *rplA* | LSU ribosomal protein L1P |
| **CBU_0228** | *rplJ* | LSU ribosomal protein L10P |
| **CBU_0229** | *rplL* | LSU ribosomal protein L12P (L7/L12) |
| **CBU_0230** |  | hypothetical protein |
| **CBU_0239** | *rplD* | LSU ribosomal protein L1E (= L4P) |
| **CBU_0434** |  | hypothetical cytosolic protein |
| **CBU_0465** |  | hypothetical protein |
| **CBU_0516** |  | hypothetical protein |
| **CBU_0537** |  | hypothetical cytosolic protein |
| **CBU_0627** |  | hypothetical protein |
| **CBU_0676** |  | UDP-glucose 4-epimerase |
| **CBU_0677** |  | NAD dependent epimerase/dehydratase family |
| **CBU_0678** |  | D-glycero-D-manno-heptose-1-phosphate adenylyltransferase |
| **CBU_0805** |  | hypothetical protein |
| **CBU_0806** |  | hypothetical protein |
| **CBU_0911** |  | hypothetical protein |
| **CBU_0981** |  | hypothetical protein |
| **CBU_0994** |  | hypothetical protein |
| **CBU_1004** | *bioC.2* | biotin synthesis protein |
| **CBU_1006** | *bioF* | 8-amino-7-oxononanoate synthase |
| **CBU_1007** | *bioB* | biotin synthase |
| **CBU_1008** | *bioA* | adenosylmethionine-8-amino-7-oxononanoate aminotransferase |
| **CBU_1145** |  | hypothetical protein |
| **CBU_1323a** |  | hypothetical cytosolic protein |
| **CBU_1426** |  | hypothetical protein |
| **CBU_1463** |  | hypothetical protein |
| **CBU_1472** |  | hypothetical protein |
| **CBU_1477** | *ahpC* | peroxiredoxin |
| **CBU_1478** | *ahpD* | peroxiredoxin reductase (NAD(P)H) |
| **CBU_1581** |  | hypothetical protein |
| **CBU_1716b** |  | hypothetical protein |
| **CBU_1824** |  | hypothetical protein |
| **CBU_1841** | *pth* | peptidyl-tRNA hydrolase |
| **CBU_1894** |  | hypothetical cytosolic protein |
| **CBU_2001** |  | hypothetical protein |
| **CBU_2003a** |  | hypothetical protein |
| **CBU_2007** |  | hypothetical protein |
| **CBUA0017** |  | hypothetical protein |
| **CBUA0022** |  | hypothetical protein |
