# Supplementary material for: Coxiella burnetii replicates in Galleria mellonella hemocytes and transcriptome mapping reveals in vivo regulated genes
Source: Virulence. 2020 Sep 24;11(1):1268–78. doi: 10.1080/21505594.2020.1819111 (PMC7549970; doi:10.1080/21505594.2020.1819111)
Supplement: Supplemental Material [file KVIR_A_1819111_SM6611.zip › Supplementary Table S8_v3.docx]

**Supplementary Table S8.** Comparison of the significantly regulated *C. burnetii* genes in *G. mellonella*, BGM cells and mice. Values in each column of *G. mellonella* indicates the number of significantly regulated genes (orange circles) shown in the top right and in the bottom left quartet (significantly upregulated and significantly downregulated, respectively) in the corresponding graphs in Supplementary Figure S7.

| ***in vitro* (ACCM-2) vs** | | ***G. mellonella*** | | | | | | | |
| --- | --- | --- | --- | --- | --- | --- | --- | --- | --- |
|  |  | **1-day p.i.** | | **2-day p.i.** | | **3-day p.i.** | | **4-day p.i.** | |
| **BGM cells (stationary phase)** | **Significantly upregulated (469)** | 158 | 33.7% | 115 | 24.5% | 105 | 22.4% | 86 | 18.3% |
|  | **Significantly downregulated (242)** | 115 | 47.5% | 81 | 33.5% | 75 | 31.0% | 69 | 28.5% |
| **Mice (20 days post-infection, spleen)** | **Significantly upregulated (332)** | 114 | 34.3% | 88 | 26.5% | 78 | 23.5% | 63 | 19.0% |
|  | **Significantly downregulated (122)** | 74 | 60.7% | 55 | 45.1% | 53 | 43.4% | 46 | 37.7% |
