# Supplementary material for: Coxiella burnetii replicates in Galleria mellonella hemocytes and transcriptome mapping reveals in vivo regulated genes
Source: Virulence. 2020 Sep 24;11(1):1268–78. doi: 10.1080/21505594.2020.1819111 (PMC7549970; doi:10.1080/21505594.2020.1819111)
Supplement: Supplemental Material [file KVIR_A_1819111_SM6611.zip › Supplementary Table S9_v3.docx]

**Supplementary Table S9.** Comparisons of the significantly upregulated and significantly downregulated transcriptomes in BGM cells or mice to the significantly regulated transcriptomes in *G. mellonella* by regression analysis. Results refer to corresponding graphs in Supplementary Figure S7.

|  | **R^2^ of linear regression** | | | |
| --- | --- | --- | --- | --- |
|  | **1d p.i.** | **2d p.i.** | **3d p.i.** | **4d p.i.** |
| **BGM vs** | 0.00043 | 0.500492 | 0.684898 | 0.889428 |
| **mice vs** | 0.000108 | 0.000023 | 0.000104 | 0.000176 |
